# Supplementary material for: Modeling poliovirus replication dynamics from live time-lapse single-cell imaging data
Source: Sci Rep. 2021 May 5;11:9622. doi: 10.1038/s41598-021-87694-x (PMC8100109; doi:10.1038/s41598-021-87694-x)
Supplement: Supplementary file 1 — Supplementary Information. [file 41598_2021_87694_MOESM1_ESM.pdf]

# Supplemental material: Modeling poliovirus replication dynamics from live time-lapse single-cell imaging data

Ashley I. Teufel, Wu Liu, Jeremy A. Draghi, Craig E. Cameron, and Claus O. Wilke

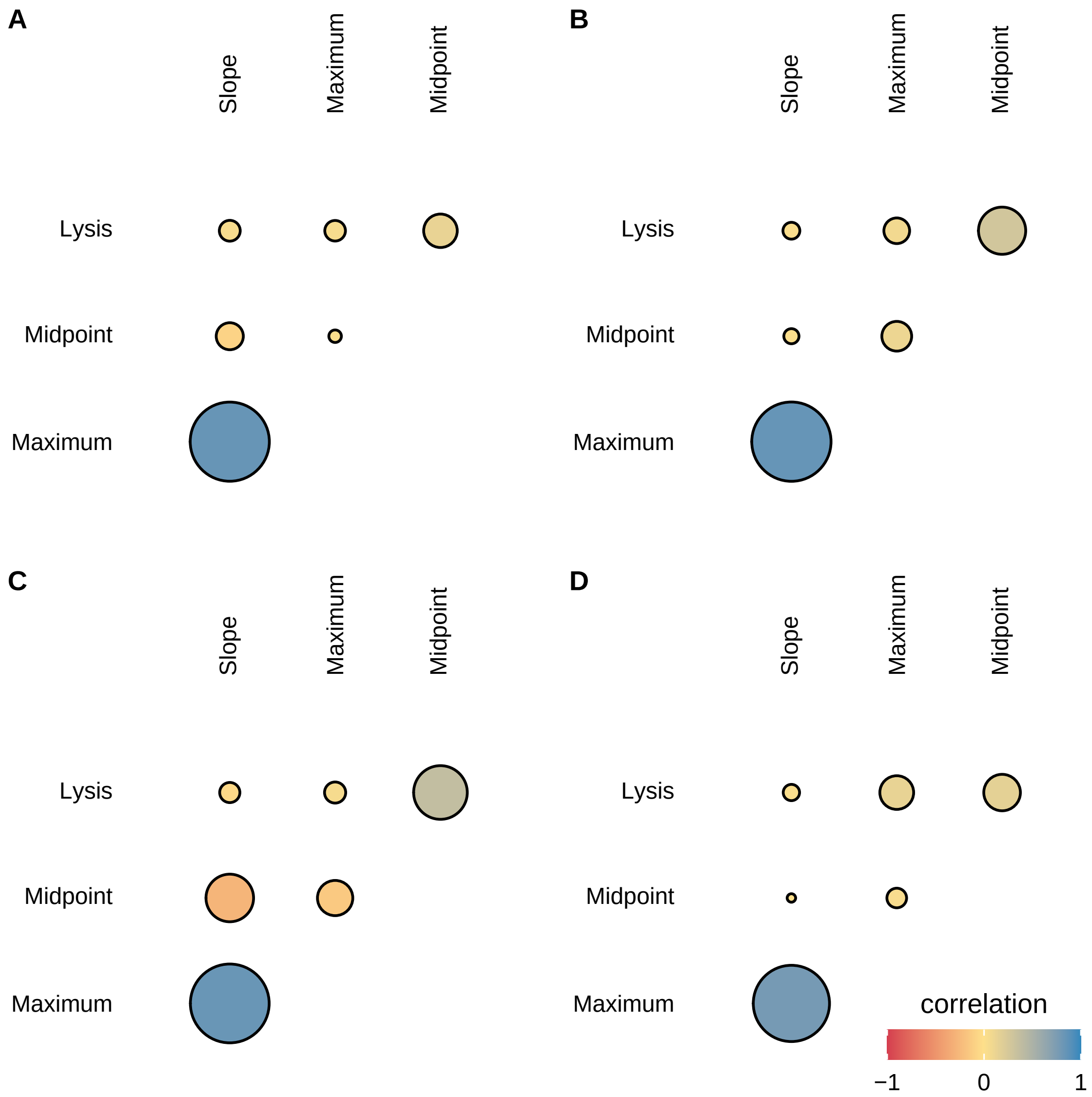


Figure S1: Correlation among the slope, maximum, midpoint, and lysis parameters in the experimentally observed data. (A) Correlations among parameters generated in the no drug experiments. (B) Correlations among parameters under treatment with rupintrivir. (C) Correlations among parameters under treatment with 2'-C-meA. (D) Correlations among parameters under treatment with ganetespib.

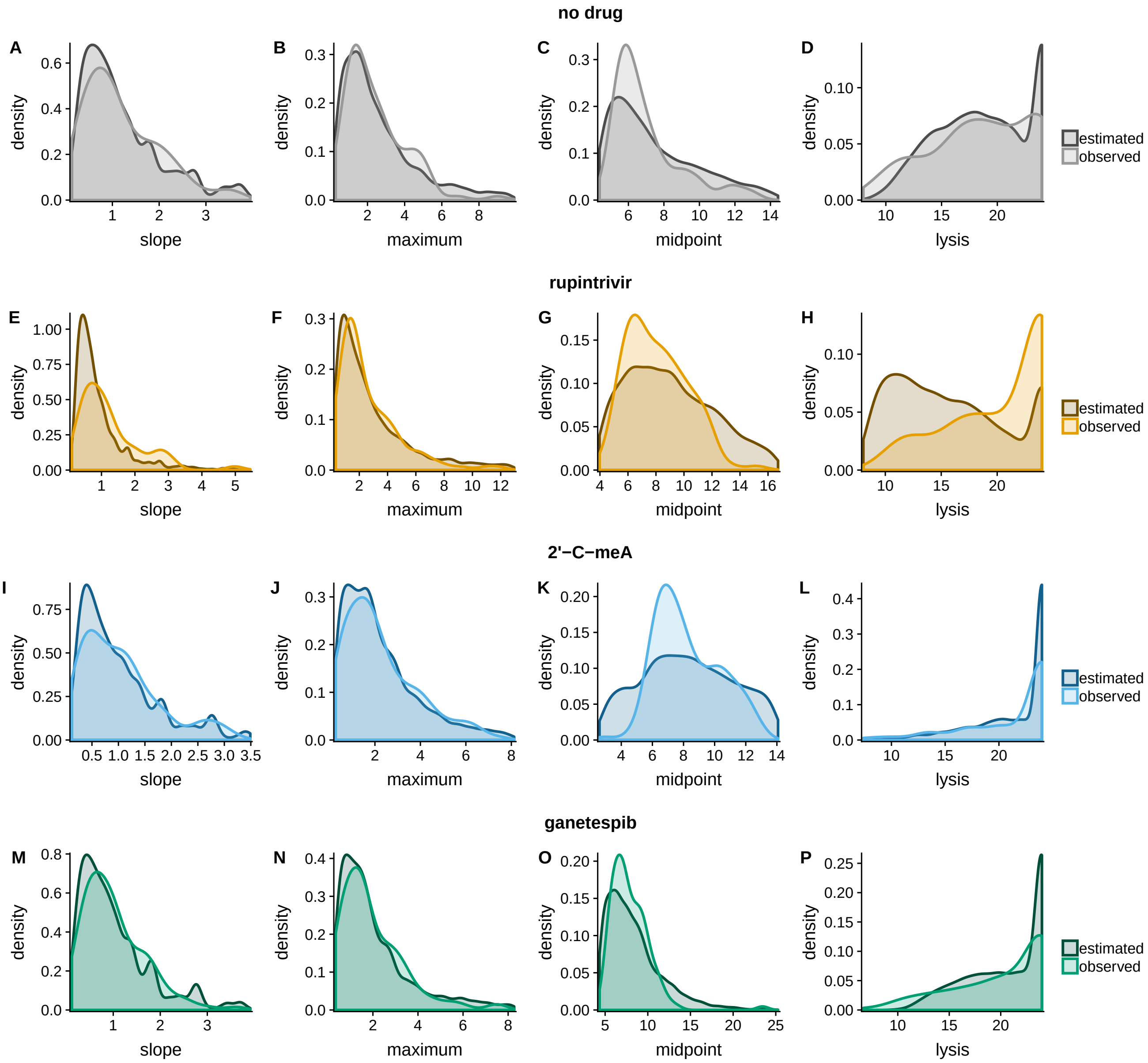


Figure S2: Visual comparison of estimated and experimentally observed distributions. (A–D) Results for experiments performed in the absence of drug treatment. (E-H) Results for experiments performed under treatment with rupintrivir. (I–L) Results for experiments performed under treatment with 2'-C-meA. (M-P) Results for experiments performed under treatment with ganetespib.

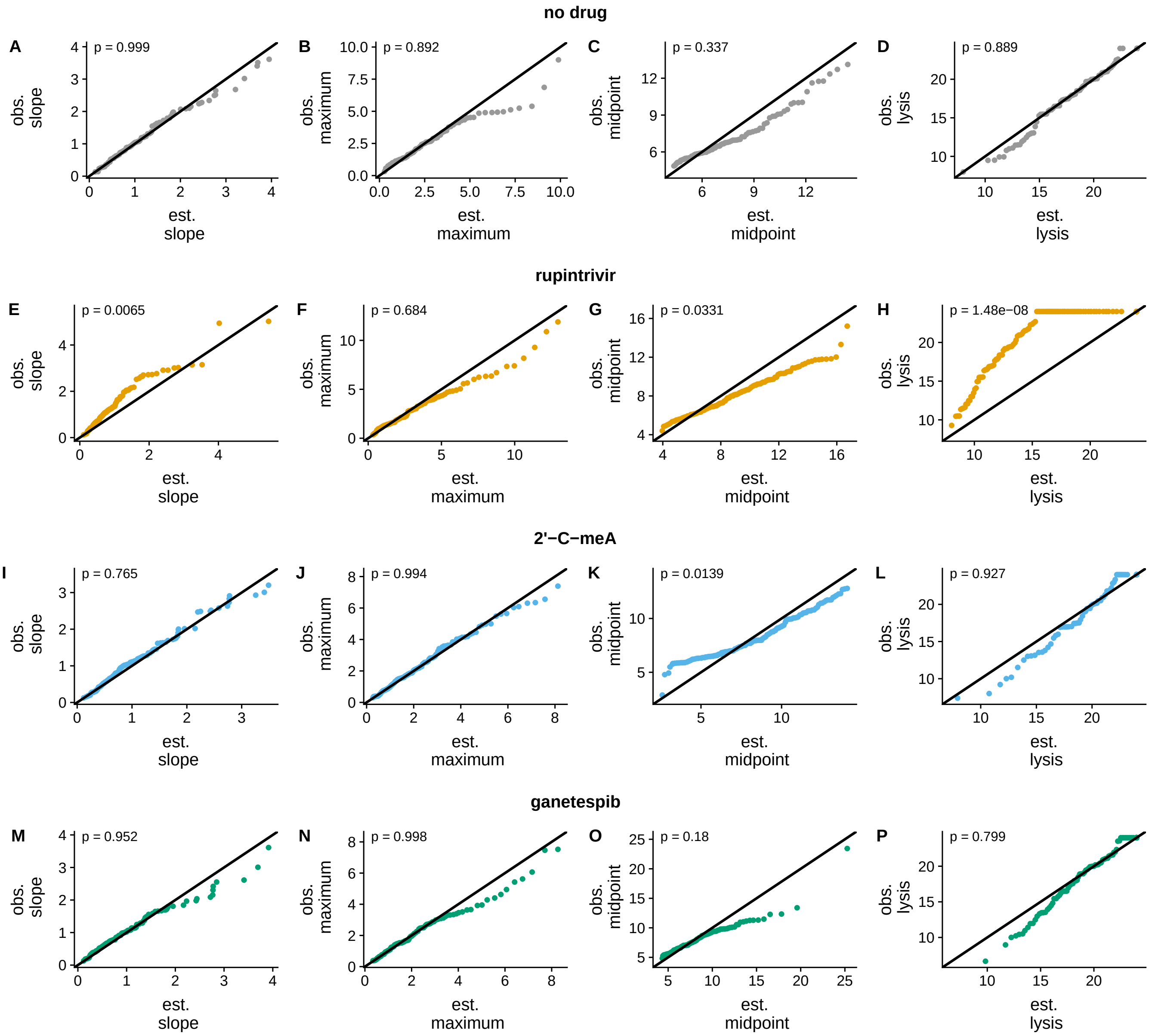


Figure S3: Quantile–quantile plots comparing estimated and observed distributions. $p$-values denote the results from K-S tests. (A–D) Results for experiments performed in the absence of drug treatment. (E-H) Results for experiments performed under treatment with rupintrivir. (I–L) Results for experiments performed under treatment with 2'-C-meA. (M-P) Results for experiments performed under treatment with ganetespib.

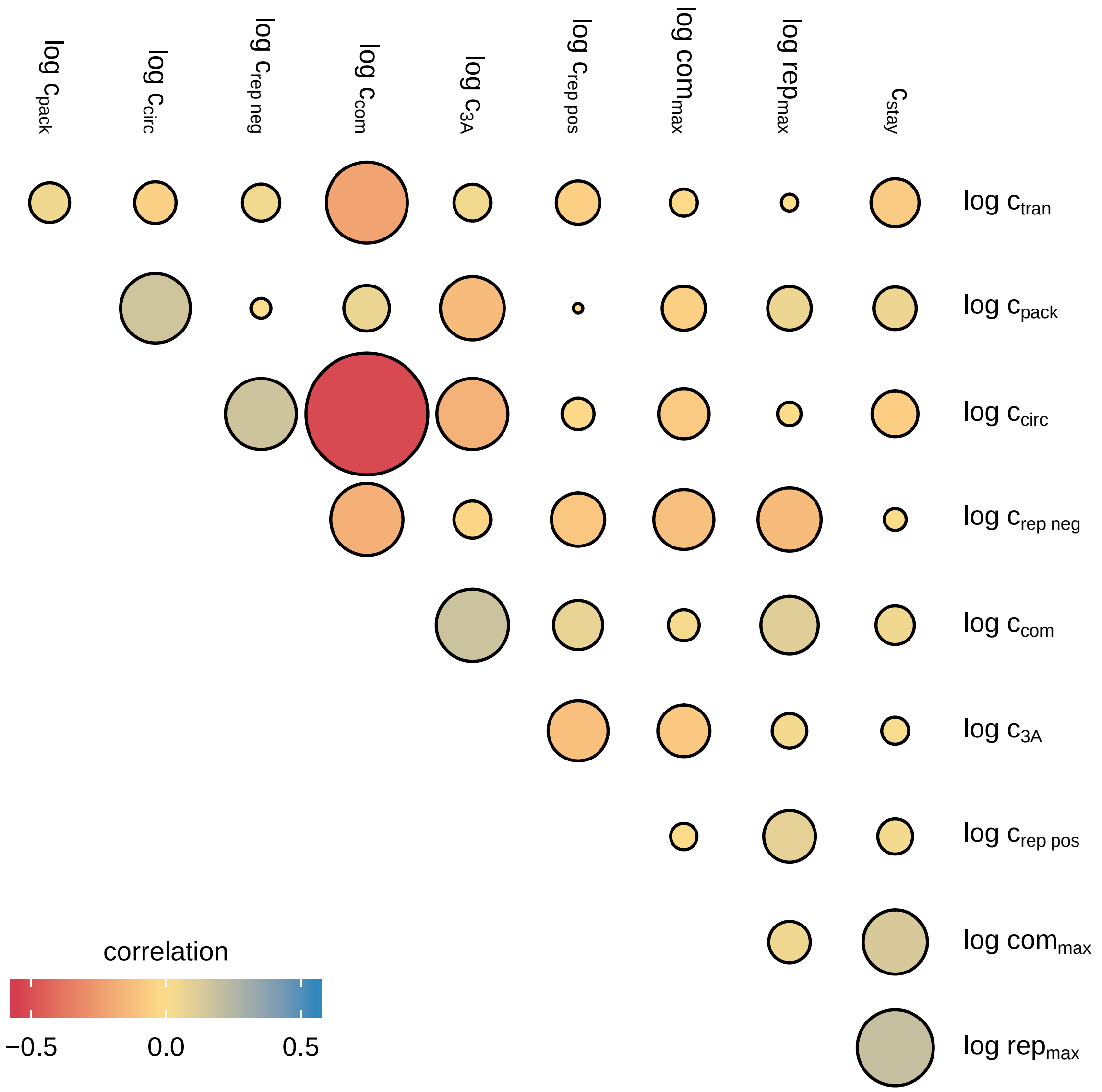


Figure S4: Correlations among parameter estimates from our mechanistic model of PV infection when the model is fit to the experimental data given in Fig. 1C-F.

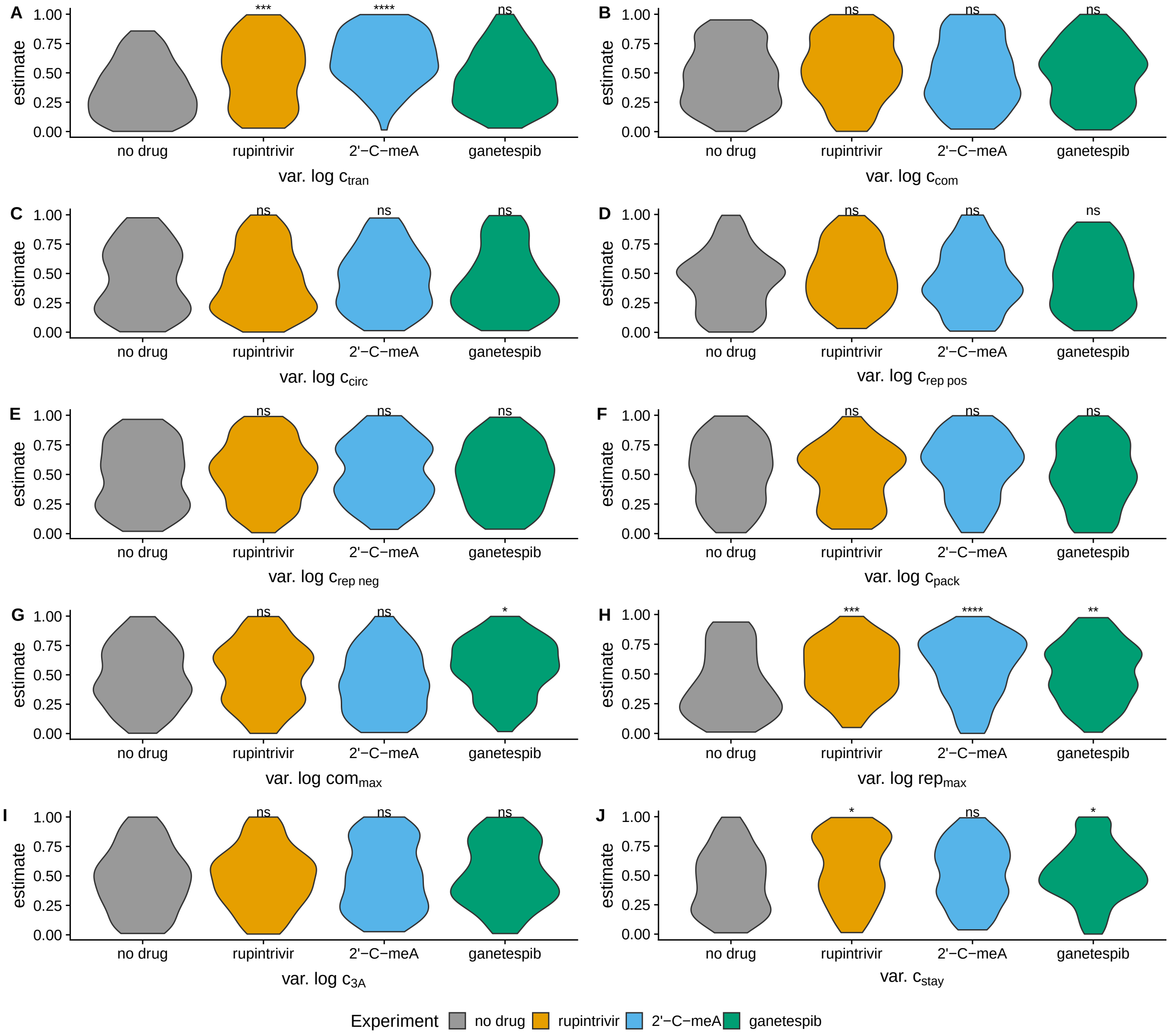


Figure S5: Comparison of posterior distributions for variance parameters between the no drug treatment and drug treatments. Asterisks indicate distributions that differ significantly from the no drug treatment. Non significant (ns) corresponds to $p > 0.05$, a single asterisk corresponds to $p \leq 0.05$, two asterisks correspond to $p \leq 0.01$, three correspond to $p \leq 0.001$, and four correspond to $p \leq 0.0001$ from a K-S test. Parameters are estimated by fitting the model described by the equations in Table 1 and illustrated in Fig. 2A. Parameters correspond to those labeled in each reaction. (A) Translation, which occurs in step 3 of the model. (B) Compartmentalization, a part of step 4. (C) Circularization, step 5. (D, E) Replication of positive and negative sense RNA, step 6. (F) Packaging, step 7. (G) The maximum number of compartments possible, considered in step 6. (H) The maximum number of replication cycles permitted by cellular resources, a limiting factor in step 4. (I) Consumption of the protein product $3A$, step 4. (J) The probability for a newly synthesized genome to stay in the replication complex, step 8.

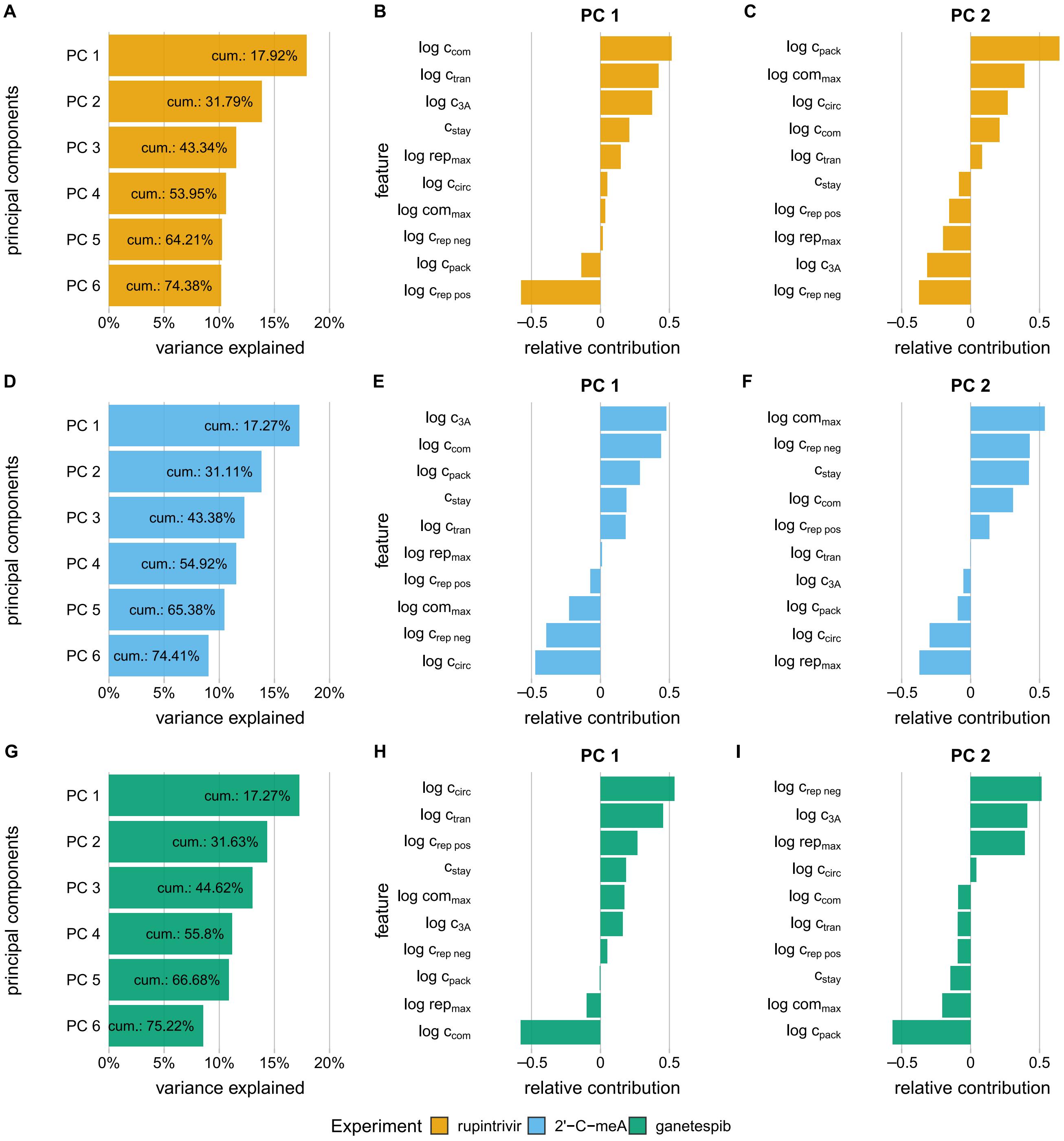


Figure S6: Principal component analysis of posterior parameter distributions estimated from fitting our mechanistic model of PV infection to experimental data generated under different drug treatments. (A) The amount of variance explained by the first 6 principal components when fitting to data generated under treatment with rupintrivir. Shown is the cumulative amount of variance explained by including each of the following components. (B, C) Relative contribution of features for the first and second principal component axis. (D) The amount of variance explained by the first 6 principal components when fitting to data generated under treatment with 2'-C-meA. Shown is the cumulative amount of variance explained by including each of the following components. (E, F) Relative contribution of features for the first and second principal component axis. (G) The amount of variance explained by the first 6 principal components when fitting to data generated under treatment with ganetespib. Shown is the cumulative amount of variance explained by including each of the following components. (H, I) Relative contribution of features for the first and second principal component axis.

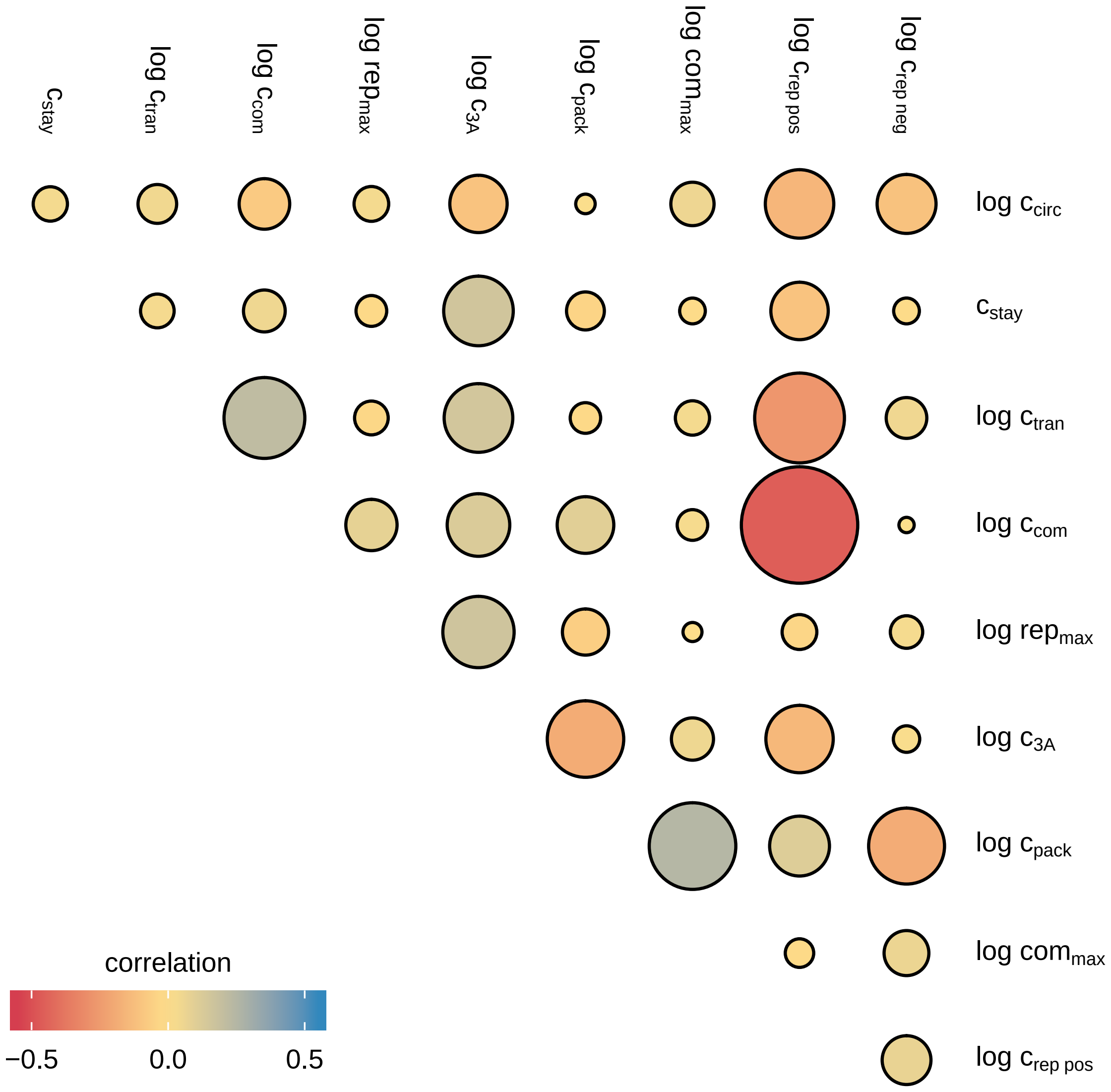


Figure S7: Correlation of parameter estimates when fitting data generated under rupintrivir treatment.

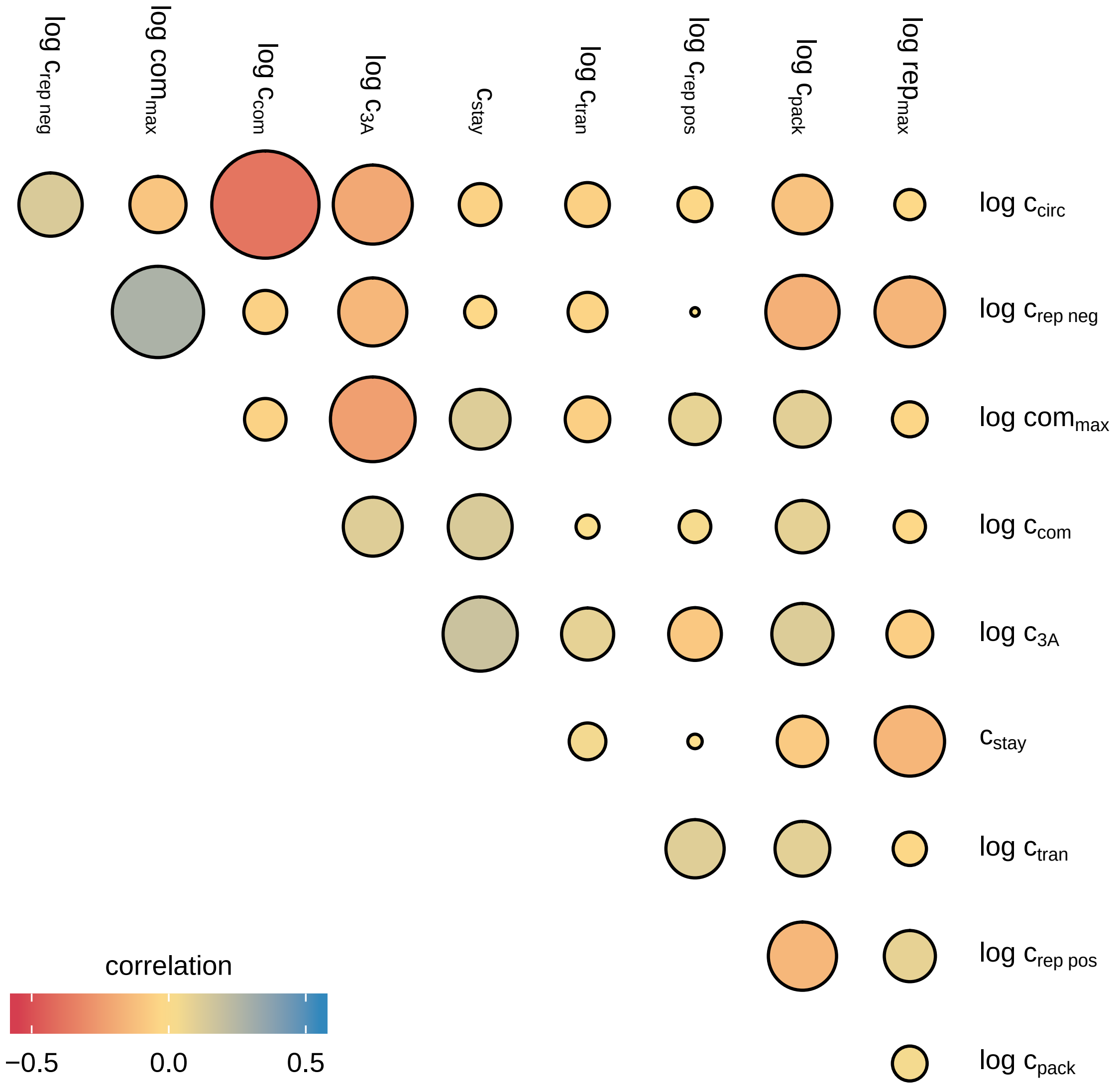


Figure S8: Correlation of parameter estimates when fitting data generated under 2’-C-meA treatment.

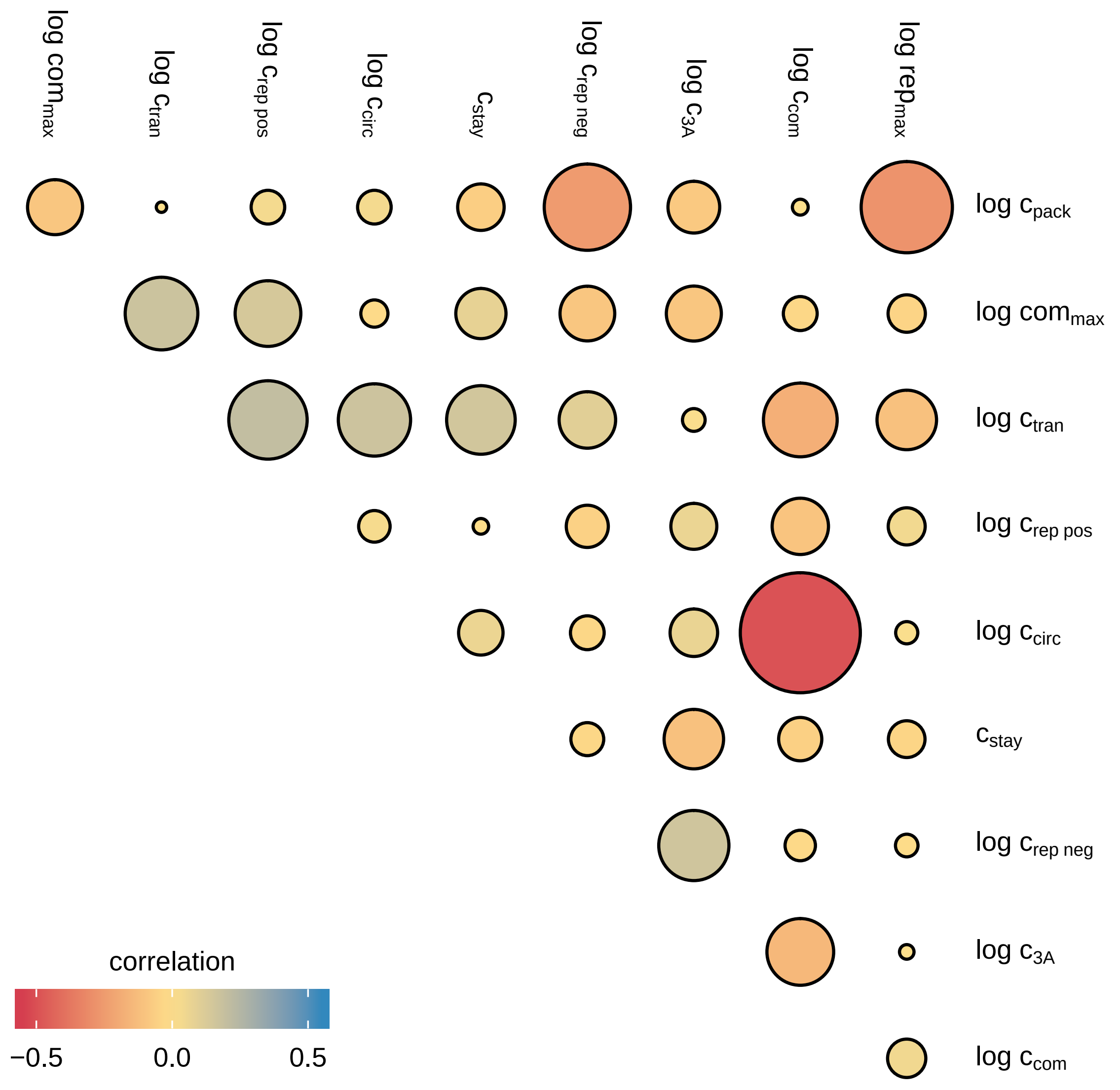


Figure S9: Correlation of parameter estimates when fitting data generated under ganetspib treatment.

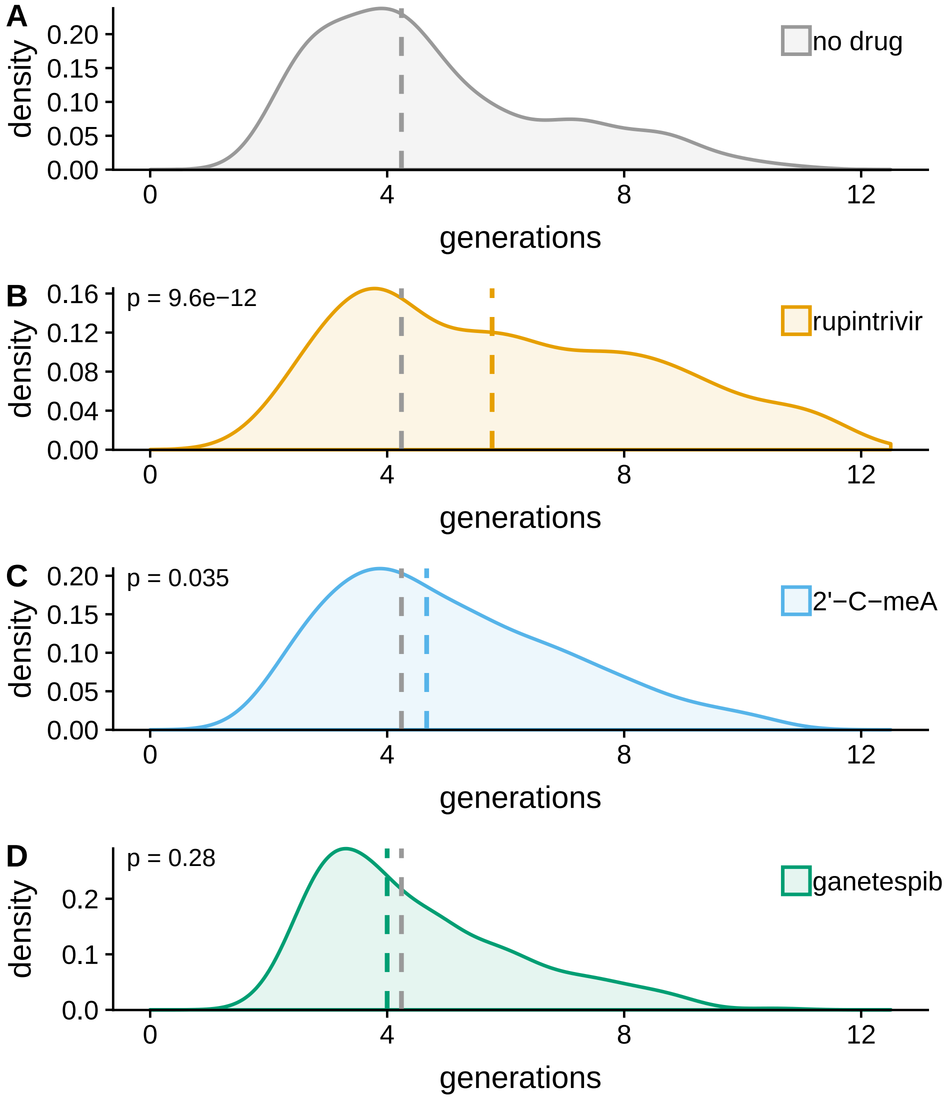


Figure S10: Comparison of mean number of generations under each of the experiments. Dashed lines denote the medians of the distributions. Gray dashed lines denote the median for the case of no drug treatment and are included in the drug treatment panels to allow for a comparison of the medians. $p$-values denote results of a K-S test to compare drug-treatment data to data generated without drug treatment. (A) Mean number of generations in the no drug case. When fitting our model to data generated without drug treatment, we estimate a median of 4.24 and a mean of 4.75 generations. (B) Mean number of generations under treatment of 2'-C-meA. Fitting our model to data generated under the drug treatment of 2'-C-meA, we estimate a median of 4.67, and a mean of 5.06 generations. (C) Mean number of generations under treatment of rupintrivir. Fitting our model to data generated under the drug treatment of rupintrivir, we estimate a median of 5.77 and a mean of 6.0 of generations. (D) Mean number of generations under treatment of ganetespib. Fitting our model to data generated under the drug treatment of ganetespib, we estimate a median of 4, and a mean of 4.5 generations.
